# Cip/Kip cyclin-dependent protein kinase inhibitors and the road to polyploidy

**DOI:** 10.1186/1747-1028-4-10

**Published:** 2009-06-02

**Authors:** Zakir Ullah, Chrissie Y Lee, Melvin L DePamphilis

**Affiliations:** 1 National Institute of Child Health and Human Development, National Institutes of Health, 9000 Rockville Pike, Bethesda, MD 20892-2753, USA

## Abstract

Cyclin-dependent kinases (CDKs) play a central role in the orderly transition from one phase of the eukaryotic mitotic cell division cycle to the next. In this context, p27^Kip1 ^(one of the CIP/KIP family of CDK specific inhibitors in mammals) or its functional analogue in other eukarya prevents a premature transition from G1 to S-phase. Recent studies have revealed that expression of a second member of this family, p57^Kip2^, is induced as trophoblast stem (TS) cells differentiate into trophoblast giant (TG) cells. p57 then inhibits CDK1 activity, an enzyme essential for initiating mitosis, thereby triggering genome endoreduplication (multiple S-phases without an intervening mitosis). Expression of p21^Cip1^, the third member of this family, is also induced in during differentiation of TS cells into TG cells where it appears to play a role in suppressing the DNA damage response pathway. Given the fact that p21 and p57 are unique to mammals, the question arises as to whether one or both of these proteins are responsible for the induction and maintenance of polyploidy during mammalian development.

## The road to polyploidy

When metazoan cells proliferate, they employ the mitotic cell cycle in which separation of sibling chromosomes during mitosis (M-phase) and DNA synthesis during genome duplication (S-phase) are separated by two intervening gaps of time called the G1 and G2-phases to generate a repeating series of events: M→G1→S→G2→M. Cell division (cytokinesis) occurs immediately after mitosis. Cell growth occurs primarily during G1-phase. In addition, metazoan cells can exit their mitotic cell cycle and enter a quiescent state termed G0 in which the living state is maintained in the absence of either cell growth or proliferation. Mitotic cell cycles restrict genome duplication to once and only once per cell division. Therefore, G1-phase somatic cells contain two copies of their genome (2N or diploid), whereas somatic cells in G2 or M-phases are tetraploid (4N DNA). Cells with greater than 4N DNA content are referred to as polyploid.

Polyploidy can result from aberrant DNA re-replication during S-phase. DNA re-replication occurs when newly assembled replication forks re-replicate parts of the genome that have already been replicated, resulting in replication bubbles within replication bubbles [1]. This occurs when one or more of the normal controls that prevent reutilization of replication origins during S-phase is circumvented. For example, DNA replication can be induced in some metazoan cells either by over-expression of Cdt1, a protein essential for loading the replicative MCM DNA helicase, or by suppression of the Cdt1 specific inhibitor geminin. Both changes promote loading of the MCM helicase at replication origins [2]. As DNA re-replication is not a normal part of mammalian growth and development, it triggers programmed cell death (apoptosis).

**Figure 1:**
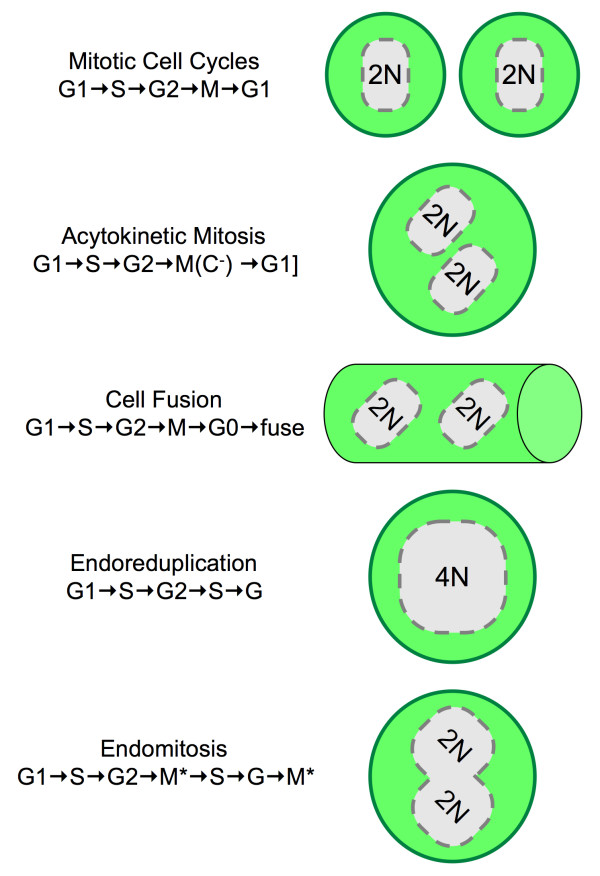
**Developmentally regulated polyploidy**. Normal mitotic cell cycles results in two diploid mononucleated daughter cells with each nucleus containing two copies of each homologous chromatid (2N). Re-replication of DNA during S-phase is an aberrant event that produces giant nuclei and apoptosis. However, developmental signals can induce cells to become polyploid either by completing mitosis in the absence of cytokinesis [(C^-^), acytokinetic mitosis], or by melding two G0-phase cells into a single cell containing two G-phase nuclei (cell fusion), or by arresting cells in G2-phase and then inducing another S-phase (endoreduplication), or by arresting cells in M-phase (M*) in the absence of cytokinesis (endomitosis). Multiple rounds of acytokinetic mitosis produce multinucleated giant cells. Multiple cell fusion events produce multinucleated myotubes in skeletal muscle. Multiple rounds of endoreduplication (endocycles) produce mononucleated giant cells, whereas multiple rounds of endomitosis produce a single multilobular nucleus.

Polyploidy can also occur as a normal component of animal or plant development. This is common among ferns, flowering plants, arthropods, fish, and salamanders, but it is rare among mammals. Developmentally programmed polyploidy is the result of multiple S-phases in the absence of cytokinesis under conditions that prevent the induction of apoptosis. Such cells are terminally differentiated, they grow in size, but they no longer proliferate. Thus, the simplest mechanism by which cells become polyploid is acytokinetic mitosis, repeated S and M phases in the absence of cytokinesis (Fig. 1). This occurs during liver development to produce multinucleated hepatocytes (Table 1, [3-19]). Multinucleated cells also arise by cell fusion, a process in which G0-phase cells simply fuse their membranes together, to produce a single cell in which multiple nuclei are distributed throughout the cytoplasm. This occurs during skeletal muscle development.

**Table 1:** Polyploidy and expression of p57 and p21 in mice

**Tissue**	**p57**	**p21**	**Polyploid**	**Mechanism**
**Placenta **[18]	+	+	+	Endoreduplication
trophoblast stem cell→giant cell				

**Bone Marrow **[4]	-	+	+	Endomitosis
megakaryoblast→megakaryocyte				

**Liver **[3]	+	+	+	Acytokinetic Mitosis
hepatocyte development				

**Skeletal Muscle **[13]	+	+	+	Cell Fusion
myoblast→myotube				

**Bone **[15]	?	+	+	Cell Fusion
monocytes→osteoclasts				

**Placenta **[8,14]	?	+	+	Cell Fusion
Syncytiotrophoblasts				

**Heart Muscle **[5,6,19]	+	+	+*	?
cardiomyocytes→myotube				

**Skin **[9]	+	+	+*	?
basal epithelial cell→keratinocyte				

**Kidney **[10]	+	-	+*	?
primitive podocyte→mature podo				

**Eye **[7,11]	+	?	?	
lens epithelial cell→fiber cell				

**Bone **[12]	+	+	?	
osteoblast→bone				

**Cartilage **[16,17]	+	?	?	
Chondrocytes→fiber cell				

→ Indicates expression of p57 and p21 up-regulated during normal cell differentiation.

* Polyploidy results from injury (e.g. heart attack) or infection.

The remaining two mechanisms by which cells become polyploid are genome endoreduplication and endomitosis. Endoreduplication occurs when a cell undergoes multiple S-phases without entering mitosis and without undergoing cytokinesis. This results in a giant cell with a single giant nucleus. Examples of endoreduplication are found among protozoa, arthropods, mollusks and plants. In contrast with these organisms, developmentally regulated endoreduplication in mammals is rare. The clearest example of endoreduplication occurs during differentiation of trophoblast stem cells into the trophoblast giant cells that are required for implantation of blastocysts into the uterine endothelium and placental development [20]. The DNA content of these giant cells generally ranges from 8N to 64N, although levels as high as 1000N have been reported

Endomitosis is similar to endoreduplication. Whereas endoreduplication results from arresting cells in G2-phase before they enter mitosis, endomitosis results from arresting cells within M-phase before they complete mitosis. The clearest example of endomitosis occurs in the bone marrow when megakaryoblasts differentiate into megakaryocytes, the cells that give rise to blood thrombocytes (platelets). The result is a single giant cell containing a single multilobulated nucleus with a genome content of up to 32N; each lobe presumably containing a diploid genome. With time, individual lobes may separate from one another to produce a multinucleated cell.

The pathways that regulate mitotic cell cycles are now understood in considerable detail [21]. This allows one to address the question, "How do mammalian cells switch from mitotic cell cycles to mechanisms that result in terminally differentiated polyploid cells?"

### Preventing Polyploidy During Mitotic Cell Cycles

Mammals encode as many as ten different cyclin-dependent kinases (CDKs), but only two of them are critical for regulating mitotic cell cycles. CDK1 activity triggers the transition from G2 to M-phase during cell proliferation, and CDK2 activity triggers the transition from G1 to S-phase. All eukarya contain an analogue of CDK1, the only CDK that is essential for mammalian development. None of the other CDKs can substitute for CDK1, and only CDK1 can substitute for CDK2 in activating S-phase in mitotic cell cycles [22-27].

DNA replication in mammalian cells begins with the assembly of prereplication complexes (preRCs) at replication origins distributed throughout the genome [28]. First, a six subunit origin recognition complex (ORC) binds to chromatin during the transition from mitotic anaphase to G1-phase [29,30]. As cells transit from mitotic telophase to G1-phase, the ORC:chromatin complex recruits Cdc6 and then Cdt1, two proteins that are required to load the six subunit MCM DNA helicase onto chromatin [31]. Additional proteins are then added to form a preinitiation complex that is acted upon by Cdk2:cyclin E(CcnE) and by DDK (a Dbf4-dependent protein kinase) to initiate DNA synthesis (S-phase) [32]. The MCM helicase is responsible for initiating DNA unwinding at the replication origin, and it continues to unwind DNA as it travels with the replication forks during S-phase.

PreRC assembly in all eukarya occurs only in the absence of CDK activity, and CDK activity is absent only during the period from mitotic anaphase to late G1-phase. From the period of late G1-phase through mitotic metaphase, CDK activity prevents assembly of new preRCs and helps to disassemble preRCs that formed during the anaphase to G1-phase transition [33]. In this way, the mitotic cell cycle prevents re-replication of the same DNA sequences from occurring before mitosis is complete and a nuclear membrane is present. The primary targets of CDK phosphorylation within the preRC are the Orc1 subunit, Cdc6 and Cdt1. Once phosphorylated these proteins are targeted for degradation by the SCF^Skp2 ^ubiquitin ligase. The resulting polyubiquitinated proteins are then degraded by the 26S proteasome. In addition, unphosphorylated Cdt1 is a substrate for the CDR^Ddb1 ^ubiquitin ligase. Both the SCF and CDR ubiquitin ligases are active only during S and G2-phases. As if this were not sufficient, multicellular eukarya contain one more mechanism for preventing loading of the MCM helicase. Cdt1 is specifically inhibited by binding to geminin, a unique metazoan protein expressed only during S, G2 and early M-phases. Consequently, both geminin and Cdk2:cylin A(CcnA) activity are required to prevent DNA re-replication in mammalian cells [34].

**Figure 2:**
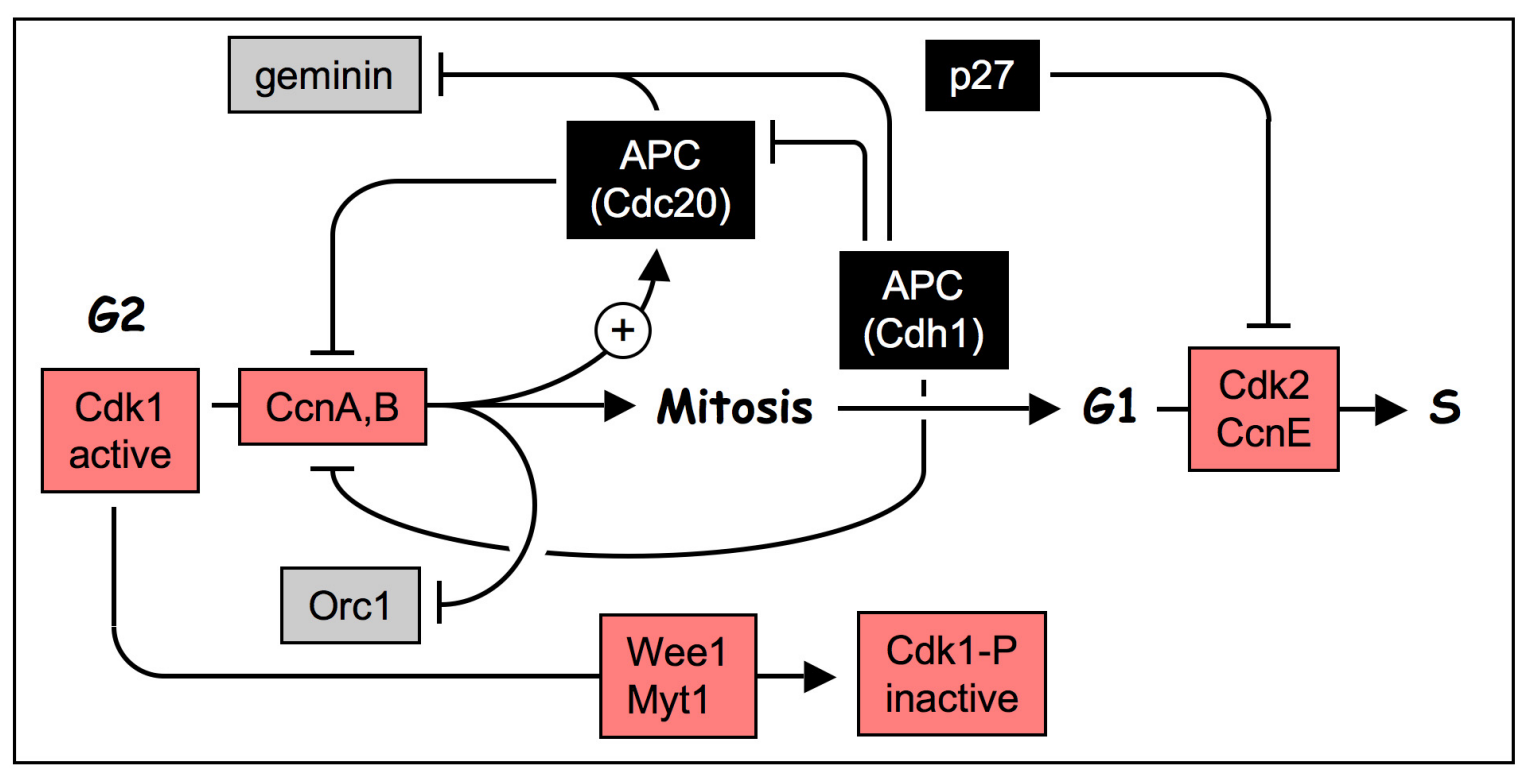
**The role of p27^Kip1 ^in preventing premature activation of S-phase**. Cdk1:CcnA and Cdk1:CcnB activities are both required to induce mitosis during mammalian cell proliferation. Either activity is required to activate the APC^Cdc20 ^ubiquitin ligase which, in turn, targets the mitotic cyclins A and B for ubiquitin mediated degradation by the 26S proteasome. Cdk1:CcnA also phosphorylates the Orc1 subunit and thereby prevents ORC binding to chromatin until metaphase is completed and APC^Cdc20 ^inhibits Cdk1 activity by reducing the pools of mitotic cyclins A and B. Once this occurs, and the spindle checkpoint releases APC^Cdc20^, cells move into anaphase and APC^Cdc20 ^targets geminin for degradation. In the absence of CDK activity, Orc1 and Cdc6 are dephosphorylated and bind to chromatin. In the absence of geminin, Cdt1 loads the MCM DNA helicase onto ORC•Cdc6•chromatin sites. Cdk1 protein is inactivated by the Wee1 and Myt1 protein kinases. APC^Cdh1 ^appears and targets Cdc20 for ubiquitin-dependent degradation. The cells are now maintained in G1-phase by APC^Cdh1 ^preventing the synthesis of cyclins A and B. However, cyclin E is not an APC substrate. As cyclin E appears, Cdk2:CcnE activity activates the replication complexes and drives the cells into S-phase. The role of p27 is to prevent premature accumulation of Cdk2:CcnE activity.

Suppression of CDK activity and activation of preRC assembly begins when the anaphase promoting complex (APC), a ubiquitin ligase, targets the mitotic cyclin A (CcnA) and cyclin B (CcnB) for ubiquitin-dependent proteolysis by the 26S proteasome (Fig. 2) [35]. This event cannot occur until several APC subunits, including its substrate targeting subunit Cdc20, have been phosphorylated by Cdk1:CcnA or Cdk1:CcnB. Even then, APC^Cdc20 ^is held back by the spindle checkpoint until metaphase is complete. Once the APC becomes active, not only are the mitotic cyclins degraded, thereby suppressing CDK1 activity and allowing the homologous chromatids to separate (anaphase), but geminin is also degraded, thereby allowing preRC assembly to begin.

Once the mitotic cyclins are degraded, CDK1 protein is converted into an inactive form through site-specific phosphorylation by the Wee1 and Myt1 protein kinases. To insure that conditions in G1-phase remain suitable for preRC assembly, two additional events occur during the M to G1-phase transition. As Cdk1 activity disappears, the APC^Cdc20 ^is dephosphorylated and therefore inactivated. In its place, the APC core subunits recruit a second substrate targeting protein called Cdh1. APC^Cdh1 ^has the same substrate specificities as APC^Cdc20^, except that APC^Cdh1 ^functions only in the absence of CDK activity, whereas APC^Cdc20 ^functions only in the presence of CDK activity. Therefore, expression of the mitotic cyclins and geminin continues to be suppressed.

### p27^Kip1 ^Prevents Premature Entrance Into S-Phase During Mitotic Cell Cycles

The second event is the appearance of the CDK-specific inhibitor p27 (Fig. 2). All mammals encode three proteins (p21^Cip1^, p27^Kip1 ^and p57^Kip2^) that belong to the CIP/KIP family and specifically inhibit cyclin-dependent protein kinases, primarily CDK1 and CDK2. One member of this CIP/KIP family, p27 [or its analogue in budding yeast (Sic1), fission yeast (Rum1), flies (Dacapo), and frogs (Xic1)], delays the transition from G1 to S-phase until assembly of prereplication complexes (preRCs) at replication origins is complete. It does so by inhibiting CDK2:CcnE activity. When cells exit mitosis, cyclin E begins to appear. Since cyclin E is not a substrate for APC^Cdh1^, it can activate CDK2. Therefore, the primary role of p27 is to delay the accumulation of Cdk2:CcnE activity until preRC assembly is complete. With time, phosphorylation of p27 by Cdk2:CcnE converts p27 into a substrate for SCF^Skp2 ^and subsequent proteolysis, thus releasing cells into S-phase. Premature activation of CDK activity results in premature entrance into S-phase with the consequence that too few replication origins are activated [36,37]. This means that replication must travel longer distances, thereby increasing the probability of replication fork malfunction and genomic instability.

### p57^Kip2 ^Triggers Endoreduplication in Trophoblast Stem Cells

**Figure 3:**
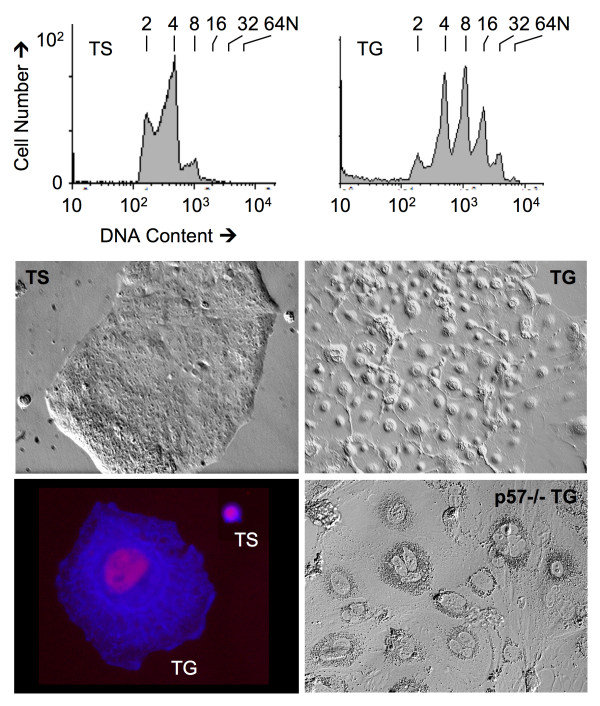
**Fluorescence activated cell sorting (FACS) analysis of mouse trophoblast stem (TS) cells undergoing mitotic cell cycles or endoreduplication as they differentiate into trophoblast giant (TG) cells when deprived of FGF4**. p57-/- TS cells respond to the same conditions by forming multinucleated TG cells. Data are from [18].

Endocycles can occur only when mitosis and cytokinesis are prevented under conditions that permit assembly and subsequent activation of preRCs. Therefore, the transition from a mitotic cell cycle to an endocycle in mammals requires suppression of CDK activity with concomitant inactivation of geminin. Once preRC assembly is complete, then CDK activity must be restored in order to initiate S-phase. This series of events is then repeated several times without going through mitosis, resulting in cells with a single nucleus that contains integral multiples of 2N DNA content (Fig. 3).

**Figure 4:**
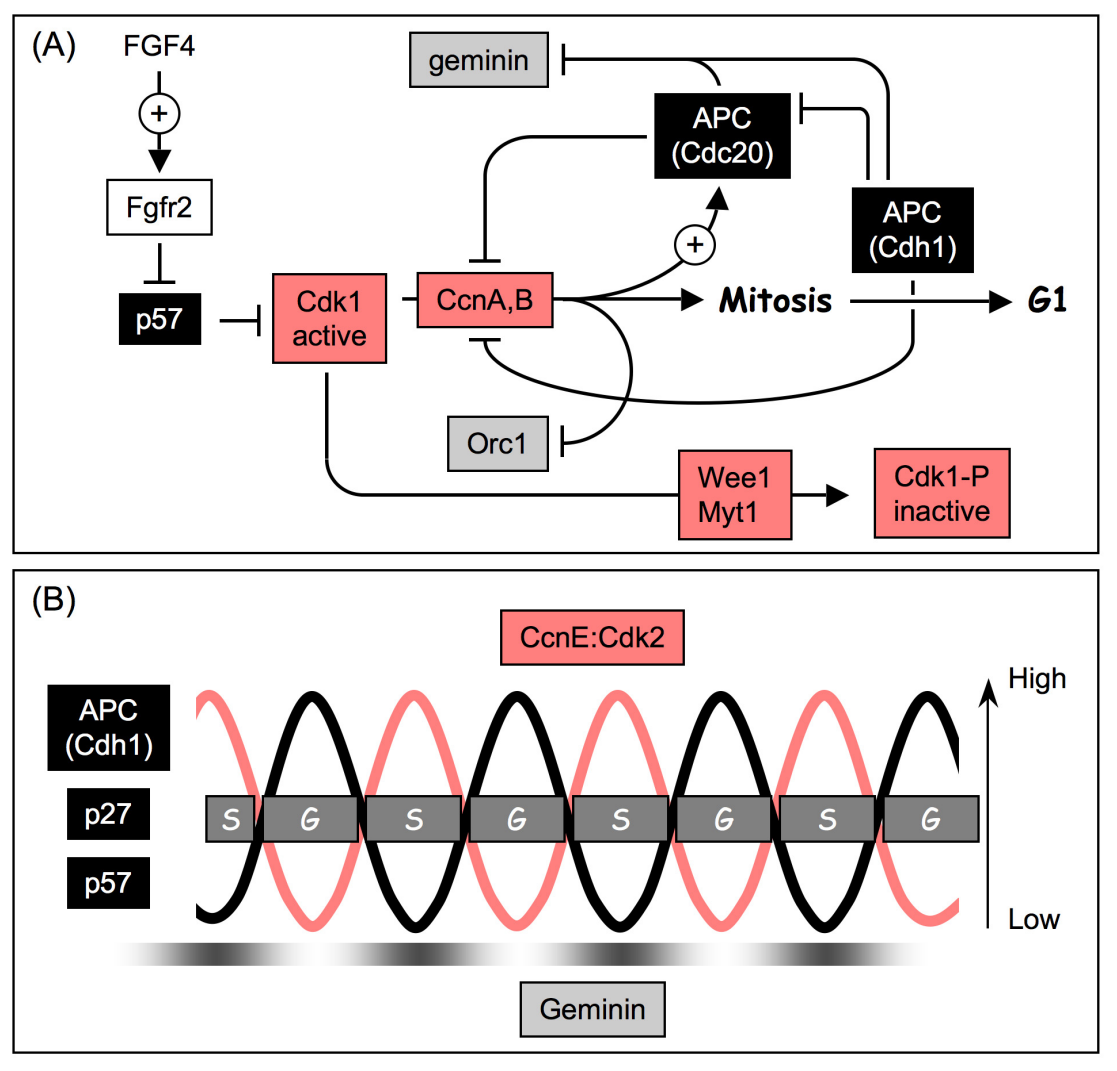
**The role of p57^Kip2 ^in the initiation and maintenance of endocycles**. **(A) **Endocycles are initiated in mouse TS cells in the absence of FGF4. When p57 is expressed in TS cells as a consequence of FGF4 deprivation, Cdk1 activity is inhibited. Consequently, APC^Cdh1 ^will be assembled instead of APC^Cdc20^. Orc1 will not be phosphorylated. In the absence of phosphorylation, any Orc1-P and Cdc6-P produced during S and G2-phases by Cdk2:CcnA will be dephosphorylated by cellular protein phosphatases. In the absence of CDK activity, APC^Cdh1 ^is assembled and targets geminin for degradation. Thus, the cell has effectively entered a G1-phase-like state without passing through mitosis. **(B) **Based on studies of endocycles in *Drosophila *follicle cells, Cdk2:cyclin E activity oscillates such that it is high during the S-phases and low during the G-phases. In contrast, APC^Cdh1/Fzr ^activity is high during G-phases and low during S-phases [40,41]. This is in keeping with the fact that Cyclin E:Cdk2 inactivates Cdh1/Fzr [51] suggesting that APC^Cdh1/Fzr ^oscillations are driven by periodic inhibition of Fzr by Cyclin E:Cdk2 [40,41]. Similarly, geminin levels are high during S-phase and low during gap phase [41]. In cells programmed for endoreduplication, inhibition of Cdk1 activity results in premature assembly of APC^Cdh1/Fzr^. Thus, a feedback loop exists that reinforces inhibition of Cdk1 activity triggered by endocycle entry. In the absence of Geminin and CDK-dependent protein phosphorylation, preRC assembly occurs as though cells had entered G1-phase. Symbols: ⊥ indicates target is inhibited, +→ indicates target is activated, → indicates product of reaction.

Recent analysis of the differentiation of mouse trophoblast stem (TS) cells into trophoblast giant (TG) cells has revealed that endoreduplication is triggered by suppression of CDK1 activity by the CDK specific inhibitor p57 [18]. Cultured TS cells undergo normal mitotic cell cycles in the presence of fibroblast growth factor 4 (FGF4), and differentiate into TG cells when deprived of FGF4, conditions that mimic trophectoderm development in vivo [38]. Differentiation of TS cells into TG cells is accompanied by genome endoreduplication with concomitant expression of both p21 and p57; cellular levels of p27 remain unchanged [18]. Of p21 and p57, only the latter is required to trigger endoreduplication and to sustain the subsequent endocycles. p57 is expressed at the end of S-phase and localized to the nucleus where it inhibits CDK1 activity and prevents entrance into mitosis (Fig. 4A). Consequently, cells accumulate in G2-phase. In the case of TS cells, they begin a new round of genome duplication for which both p57 and APC^Cdh1 ^are essential [18,39]. Recent analysis of endocycles in *Drosophila *follicle cells demonstrates that geminin activity during endocycles is regulated by APC^Cdh1/Fzr ^activity [40,41]. Thus, the role of p57 is to inhibit CDK1, one consequence of which is the appearance of APC^Cdh1 ^in place of APC^Cdc20^. The role of APC^Cdh1 ^is to inactivate geminin. The essential role of p57 in triggering endoreduplication in TS cells is the single feature that distinguishes endocycles in mammalian cells from endocycles in *Drosophila *[1].

Several lines of evidence confirm that the role of p57 during differentiation of TS cells into TG cells is to inhibit CDK1 activity [18]. The appearance of p57 was coincident with the disappearance of CDK1 activity and the appearance of endoreduplication. CDK1 co-immuno-precipitated with p57 from lysates of TG cells, but not TS cells. TS cells that lack a functional p57 gene fail to arrest mitosis and fail to endoreduplicate their genome when deprived of FGF4, although they do increase their size and express genes characteristic of TG cells. p57-/- TS cells deprived of FGF4 form multinucleated giant cells as a consequence of incomplete cytokinesis (Fig. 4). p57-/- TS cells cannot endoreduplicate their genomes, because p57 is required to inhibit CDK1. This was confirmed by the fact that RO3306, a drug that selectively inhibits CDK1 [18,42], induces endocycles in p57-/- TS cells as effectively as it does in wild-type TS cells cultured in the presence of FGF4. This observation provides an explanation for the appearance of multinucleated syncytiotrophoblasts [8]. Like p57-/- TG cells, syncytiotrophoblasts express p21, but not p57 [14], suggesting that loss of p57 activity in these trophoblast cells results in acytokinetic mitosis. Alternatively, syncytiotrophoblasts may result from cell fusion [8].

The fact that p27 is expressed at the same level in both TS and TG cells, and that p21 is also induced during differentiation of p57-/- TS cells into TG cells suggests that neither of these CDK-specific inhibitors triggers endoreduplication in response to FGF4 deprivation. In fact, endoreduplication in p21-/- TG cells is delayed until the appearance of p57. One explanation for the failure of either p21 or p27 to substitute for the role of p57 in TS cell differentiation is that neither p21 nor p27 are expressed at the same time and place as p57. However, a recent study in which the p57 gene was replaced by the p27 gene in mice suggests that this is not the case. p27 shares sequence similarity with both the N-terminal and C-terminal regions of p57. Mice containing both the normal p27 alleles as well as two additional copies of the p27 gene under control of the native p57 gene promoter revealed that many, but not all, of the defects in mouse development associated with the absence of a functional p57 gene could be rescued by p27 [43]. One tissue that did not respond to p27 therapy was the placenta. Recombinant p27 protein expressed from the p57 promoter failed to accumulate in the placenta, although the level of p27 mRNA was equivalent to that of p57 mRNA in wild-type placenta. Thus, p27 expressed from the p57 promoter is selectively degraded whereas p57 expressed from the p57 promoter is not. Moreover, KPC and Pirh2, two ubiquitin ligases that selectively target p27 for degradation [44,45], are expressed prominently in the same placental layers that express high levels of p57. Therefore, p27 can not compensate for p57 in specific regions of the placenta, which is why placental dysplasia observed in p57 null mice is not restored by replacing p57 with p27.

### p57^Kip2 ^Oscillations Sustain Endocycles

The requirement for p57 to trigger endoreduplication in TS cells by inhibiting CDK1 presents a conundrum, because p57 is also a potent inhibitor of CDK2, an enzyme that normally drives cells from G1-phase into S-phase. Just as in mitotic cell cycles, activation of S-phase requires either Cdk2 or Cdk1 activity [18]. Therefore, the cellular levels of CDK specific inhibitors must oscillate during endocycles just as they do doing mitotic cell cycles. In fact, p57 is present in TG cells only when they are not undergoing DNA replication (G-phase [18,46]). Inactivation of both p27 and p57 is achieved by site-specific phosphorylation of these two proteins by Cdk2:CcnE, one of the two protein kinases that activates preinitiation complexes to begin DNA synthesis. Cyclin E (CcnE) is required to sustain endocycles in mammals [47,48], and unlike the mitotic cyclins A and B, cyclin E is not an APC substrate [35]. The phosphorylated forms of p27 and p57 are substrates for the ubiquitin ligase SCF^Skp2 ^which targets them for proteolysis by the 26S proteasome [49,50]. This step is necessary for both mitotic cell cycles and for endocycles. If a non-degradable form of p57 that lacks the CDK-target site is expressed in Rcho-1 cells (a rat cell line that models TS cells), or if wild-type p57 is over-expressed in these cells, these cells neither proliferate nor endoreduplicate their genome [46]. The events occur after removal of FGF4 that trigger accumulation of p57 as the TS cells differentiate into TG cells remain to be determined.

Endocycles are successive oscillations of G and S-phases (Fig. 4B). In both flies and mammals, these oscillations require both CDK2:cyclin E and APC^Cdh1/Fzr ^activities [1]. Since phosphorylation of Cdh1/Fzr by CDK2:cyclin E inhibits APC^Cdh1/Fzr ^activity [51], oscillations of APC^Cdh1/Fzr ^activity appear to be driven by periodic inactivation of Cdh1/Fzr by CDK2:cyclin E [40,41]. Low CDK2:cyclin E activity during G-phases allows high APC^Cdh1/Fzr ^activity, which in turn, degrades mitotic cyclins and Geminin, thereby initiating preRC assembly. High CDK2:cyclin E activity during the G1 to S-phase transition inhibits APC^Cdh1/Fzr^, thereby initiating S-phase and allowing geminin to accumulate [41]. Geminin should prevent DNA re-replication during endocycle S-phases. CDK2:cyclin E also phosphorylates Cdc6, thereby restricting preRC assembly to the G-phase while simultaneously protecting Cdc6 from APC-dependent proteolysis [52]. Cdc6 is subsequently dephosphorylated and assembled into preRCs during the G-phase. Thus, inactivation of CDK1 results in premature expression of APC^Cdh1/Fzr ^activity which reinforces inhibition of CDK1 activity by degrading mitotic cyclins, and in addition, eliminates the block to preRC assembly imposed by geminin.

The role of p57 during endocycles, and presumably p27 as well, is to prevent CDK1:Cyclin E or CDK2:Cyclin E activities from accumulating before preRC assembly is complete. Otherwise, cells would initiate S-phase prematurely, resulting in genomic instability that would trigger apoptosis. The reiteration of these events, driven by the oscillation of CDK2:Cyclin E activity, appears to be the primary driving force behind multiple endocycles [40,41]. In mammalian mitotic cell cycles, CDK2:Cyclin A phosphorylates cyclin E, thereby enabling SCF^Skp2 ^to target cyclin E for ubiquitin-dependent degradation. Presumably, the same mechanism accounts for oscillation of CDK2:Cyclin E activity during mammalian endocycles, as well.

### A Role For p21^Cip1 ^In Production Of Polyploid Cells

**Figure 5:**
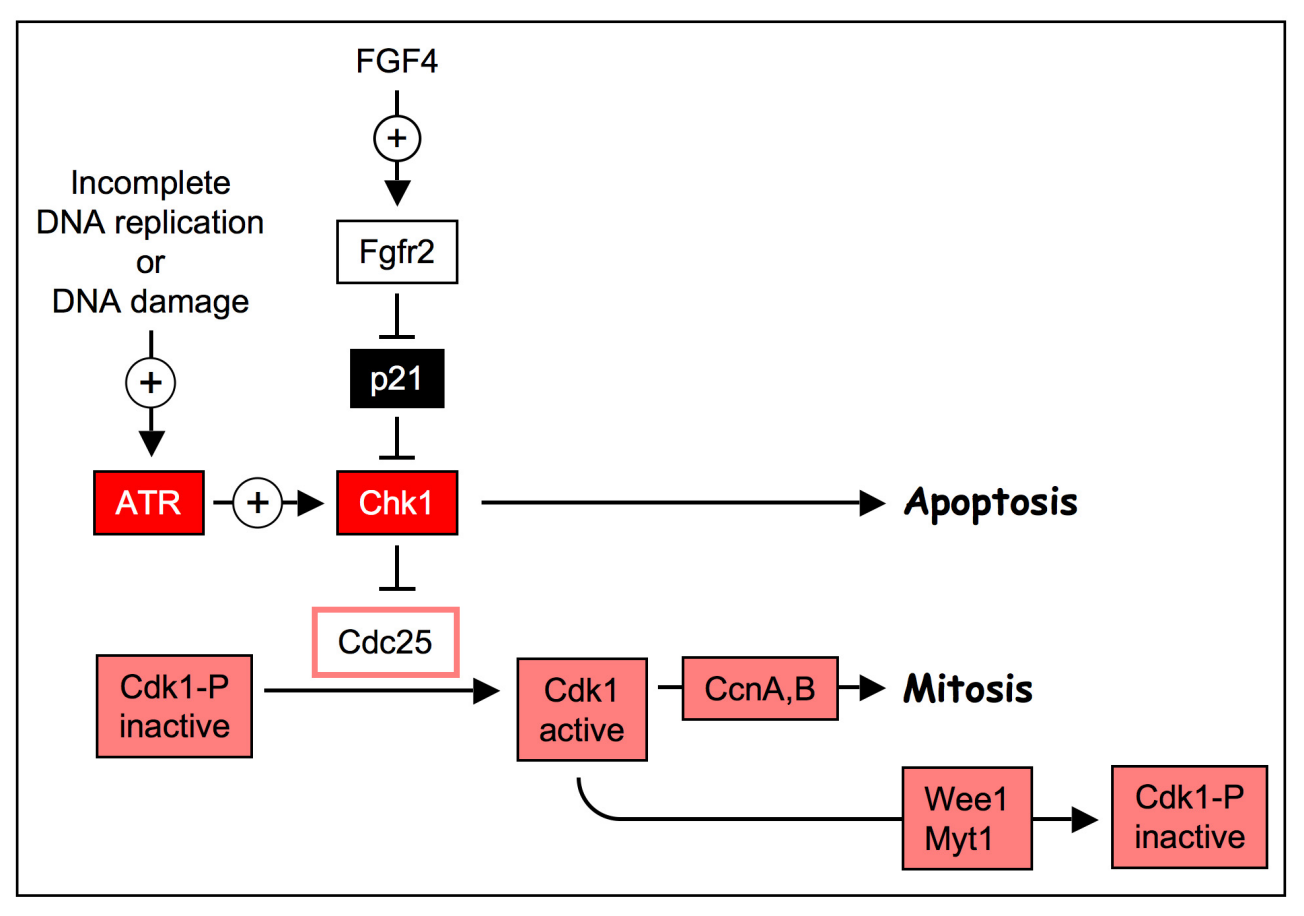
**Role of p21^Cip1 ^in preventing apoptosis during endocycles**. Upon completion of DNA replication in mitotic cell cycles, Cdc25 protein phosphatase and cyclin B are transported into the nucleus where they convert the inactive form of Cdk1 into an active form that can bind to cyclins and initiate mitosis. ATR is a protein kinase that senses the presence of excess single-stranded DNA resulting either from stalled replication forks, or from DNA damage. ATR then phosphorylates Chk1. This activates the Chk1 protein kinase and it, in turn, phosphorylates Cdc25, inhibiting it and thereby preventing cells from entering mitosis until the problem is corrected. p21 either directly or indirectly prevents expression of Chk1 protein. Under these conditions, cells do not trigger apoptosis in response to incomplete DNA synthesis or DNA damage.

One remarkable feature of cells undergoing endocycles is their ability to tolerate genotoxic stress induced either from DNA damage or incomplete DNA replication. In *Drosophila*, this apparently is due to the absence of a checkpoint that insures completion of S-phase [53-55]. In mammals, suppression of apoptosis in endocycling cells is linked to expression of the CDK-specific inhibitor p21 [18], a cyclin-dependent protein kinase inhibitor whose abundance increases in cells exposed to radiation or other DNA-damaging agents. Consequently, CDK-dependent cell cycle events are arrested until these problems can be corrected. Mice that lack p21 are hypersensitive to gamma-irradiation and exhibit a greater incidence of metastatic tumors [56]. The primary role of p21 during TS to TG cell differentiation appears to be prevention of apoptosis in response to incomplete DNA synthesis and DNA damage that may occur during endoreduplication. It does this by suppressing (either directly or indirectly) expression of Chk1, a key intermediary in the ATR-Chk1-Cdc25 DNA damage response pathway (Fig. 5). Chk1 is required for preventing proliferating cells from entering mitosis with DNA damage or stalled replication forks [57,58]. A strong correlation exists between expression of p21, suppression of Chk1, and reduced sensitivity to genotoxic stress [18]. Cells that express Chk1, such as TS and ES cells, are sensitive to genotoxic stress, whereas cells that do not express Chk1 (e.g. TG cells) are not. p21 alone can down-regulate Chk1 protein levels in cancer cells, and cancer cells lacking p21 do not suppress Chk1 in response to genotoxic stress [59]. However, TG cells lacking p21 still suppress Chk1 protein levels, but only after they express p57 [18]. Presumably, p57 can substitute for p21 in suppressing Chk1 protein synthesis.

### p21^Cip1 ^And p57^Kip2 ^Promote Cell Fusion In Myoblasts

Three examples of developmentally regulated cell fusion have been reported (Table 1). One occurs in bone marrow where monocytic macrophages differentiate into polynucleated osteoclasts, the cell responsible for bone resorption by removing its mineralized matrix. Another is syncytiotrophoblasts, multinucleated cells found in the placenta. They are the outer syncytial layer of the trophoblasts and actively invade the uterine wall. A well characterized example, however, is the differentiation of skeletal muscle myoblasts into myotubes. During myogenesis, for example, mononucleated myoblasts withdraw from the cell cycle, initiate muscle specific gene expression, and subsequently fuse with one another to form multinucleated myofibers [60]. Cell cycle arrest is an essential step in skeletal muscle differentiation. Once arrested, mononucleated myoblasts fuse to form syncytial myofibers. p21 and p57 are highly expressed in skeletal muscles and redundantly induce cell cycle arrest during differentiation. Inactivation of either p21 or p57 does not affect muscle differentiation in mice. However, inactivation of both genes produced the same muscle deficient phenotype observed with nullizygous myogenin mice [56]. Their activity is linked directly to MyoD expression, one of the earliest myogenic regulatory proteins expressed during muscle differentiation. MyoD commits mesoderm cells to a skeletal lineage, and in doing so it can block cell proliferation. MyoD is phosphorylated on Ser 200 in proliferating myoblasts by either Cdk1 or Cdk2 in myoblasts [58,59]. During differentiation MyoD induces expression p21 and p57 [61]. p21 and p57 prevent CDK-dependent phosphorylation of MyoD and thereby prevent its ubiquitin-dependent degradation. Analysis of synchronized myoblasts reveals that they exit the mitotic cell cycle during G1-phase, when MyoD levels are highest. Thus, inhibition of CDK:cyclin E activity by either p21 or p57 is likely the mechanism by which these CDK specific inhibitors trigger differentiation of myoblasts into myotubes.

### Possible Role For CIP/KIP Proteins In Endomitosis

Another role for p21 may be in triggering endomitosis during differentiation of megakaryoblasts (MKBs) into megakaryocytes (MKCs). Thrombopoietin stimulates differentiation of MKBs into MKCs with concomitant stimulation of p21 expression [62]. As with TS to TG cell differentiation, Cdk1 is degraded during MKB to MKC differentiation, p21, p27 and cyclin E levels remain high, and cyclins A and B are present, but at a reduced level [4,63,64]. However, in contrast with TG cells, MKCs express only p21 and p27; p57 has not been detected [4], suggesting that p21 may inhibit entrance into mitosis. Given the role proposed for p21 during normal mitotic cell cycles, stimulation of p21 in cells developmentally programmed for endoreduplication could have the same effect as stimulation of p57 that is to arrest mitosis.

During mitotic cell cycles, p21 accumulates transiently in late G1-phase of where it helps to prevent premature entry into S-phase by inhibiting CDK2:cyclin E activity [65], and it is then targeted for degradation by the SCF ubiquitin ligase during the G1 to S-phase transition. p21 again accumulates during G2-phase where it helps to prevent premature activation of CDK1-mitotic cyclin activities, and it is then degraded in prometaphase following its ubiquitination by APC^Cdc20 ^[66]. Thus, stimulation of p21 levels during G2-phase would provide cells with another mechanism to inhibit Cdk1 activity, arrest mitosis and initiate preRC assembly. However, mice nullizygous for p21 are viable and fertile [67] and produce MKCs [4] that contain giant nuclei indistinguishable from those in wild-type animals. Since p57 expression is not induced in these p21-/- MKCs, p27 may substitute for the role of p21 during MKB to MKC differentiation, as it appears to do so in osteoblast differentiation [68]. As discussed below, genetic analyses of mouse development and cell differentiation in vitro has revealed functional redundancies among the CIP/KIP family of proteins.

MKB differentiation is reminiscent of differentiation of p57-/- TS cells into TG cells. TS cells expressing both p57 and p21 arrest proliferation in G2-phase and endoreduplicate. However, TS cells that are nullizygous for p57 continue to divide in the absence of FGF4 until p21 is expressed, at which time they become multinucleated [18]. These results reveal that Cdk1 activity is strongly inhibited when p57 and p21 are both expressed during TS to TG cells differentiation, but only weakly inhibited when p21 alone is expressed. Therefore, when thrombopoietin stimulates p21 expression in MKBs, cells will not arrest in G2-phase (as they do when p57 is expressed), but will progress into mitosis albeit slowly. Complete inhibition of CDK1 in these cells may require additional steps.

Other mechanisms that have been suggested for induction of endomitosis in MKBs include suppression of cyclin B1 expression [69,70], suppression of CDC25C expression [71] [the protein phosphatase that converts Cdk1-P into its active form (Fig. 5)], and degradation of CDK1 [72]. One difficulty in these studies is that many of them have used either megakaryocytic leukemia cell lines, such as UT-7 and HEK, or immortalized cell lines, such as MegT, which may not accurately represent the events in differentiation of primary MKBs [62,69,72]. Obviously, further studies will be required to elucidate the mechanism of endomitosis and its relationship with endoreduplication. Whatever the mechanism, it must account for the fact that MKCs enter mitosis but do not complete it. Therefore, some CDK1:mitotic cyclin activity is needed to transit prophase, prometaphase and metaphase and the presence of this activity will be needed in successive cycles. In addition, the transition from metaphase to anaphase requires the appearance of APC activity with concomitant the loss of CDK1:mitotic cyclin activity.

### Acytokinetic Mitosis

Acytokinetic mitosis occurs when cells separate their homologous chromosomes into two groups (mitosis) but fail to form a cleavage furrow in the center of the cell and undergo cell division (cytokinesis). This occurs during postnatal liver development. Liver parenchyma undergoes dramatic changes characterized by gradual appearance of tetraploid and octoploid hepatocytes containing one or two nuclei [73]. Thus, hepatocytes are exclusively diploid in the liver of a newborn rat, but in adult rats, approximately 25% of these cells are diploid, 70% are tetraploid and 5% are octoploid. Binucleated tetraploid hepatocytes are the result of acytokinetic mitosis. The mechanism that specifically prevents cytokinesis is unknown.

TG cells in bovine and alpaca placenta can also develop two or more nuclei, indicative of cycles of acytokinetic mitoses or, in the case of alpaca placenta, lobulated nuclei suggestive of endomitosis [74]. These multinuclear TG cells are reminiscent of the multinuclear p57-/- TG cells that develop in vitro [18], suggesting that down-regulation of p57 during placental development could give rise to acytokinetic mitosis.

### Cell Differentiation

Induction of p57 appears to play a critical role in differentiation of some cell types by arresting their mitotic cell cycle without inducing polyploidy. Osteoblasts, for example, differentiate into mineralized bones. In doing so, they appear to first arrest proliferation by induction of p57, an event that can be induced in vitro by serum starvation of osteoblastic cells [12]. Events that increase p57 levels in osteoblasts stimulate differentiation into bone, whereas events that decrease p57 levels inhibit differentiation.

A second example is chrondrocyte differentiation, an event required for bone formation. Chondrocytes pass from a proliferative state to a postmitotic, hypertrophic state equivalent to G0-phase. As with osteoblasts and myoblasts, growth arrest is a central feature of chrondrocyte differentiation. Mice lacking p57 exhibit chondrodysplasia and loss of collagen type X expression. Over-expression either of p57, or of p57 and p21 together induced growth arrest in chondrocytes, but it was not sufficient for the induction of collagen type X [17]. Thus, p57-mediated growth arrest is not sufficient for expression of the hypertrophic phenotype, but rather it occurs in parallel with other aspects of the differentiation pathway. Conversely, parathyroid hormone-related peptide stimulates chondrocyte proliferation during bone development, and the effects of this mitogen are mediated, at least in part, through suppression of p57 expression [16].

Other examples are skeletal myoblasts, lens fiber cells, retinal cells, keratinocytes and intestinal epithelial cells (Table 1).

### The Special Place Of p57 In Mammalian Development

p57 is the only CIP/KIP protein that is essential for mammalian development. Compared to p21 and p27, p57 exhibits a restricted pattern of expression during embryonic development and in adult tissues. However, its expression does not always result in polyploid cells indicating that expression of p57 only in the proper context of development can result in polyploid cells. Expression of p57, on the other hand, is often associated with terminal differentiation (Table 1). In the mouse, p57 and p21 are co-expressed in cartilage and skeletal muscle during development although other tissue types (brain, skin, nose, and eyes) exhibit non-overlapping patterns of p21 and p57 [75,76]. Like p57 and p21, p27 is also expressed highly in the skeletal muscle and other tissues expressing p27 include the brain, heart, placenta, lung, kidney, and pancreas [77]. Expression of p57 appears to be restricted to terminally differentiated cells such as trophoblast giant cells, myotubes, retinal fiber cells, osteoblasts, and keratinocytes. Highest expression of p57 is observed in the placenta.

In mouse embryos, p57 protein is first detected in the heart (E10.5), and then in neural tissues (E11.5) called Rathke's pouch and the infundibulum, which become the pituitary, and some cells of the dorsal brain [19]. By day E13.5, p57 is present in cells of the heart and skeleton-muscular system, neural system (including choroid plexus), parenchymal organs such as lung and kidney, and in extraembryonic tissues of the placenta. After E13.5, p57 protein levels decline markedly in all tissues except skeletal muscle where staining persists at relatively high levels to day E17.5. In adult mice, p57 RNA is localized to the brain, eye, cartilage, skeletal muscle, kidney, heart, lung, and liver [75].

### Functional Redundancy Of CIP/KIP Proteins

CIP/KIP family members exhibit a remarkable ability to substitute for one another in various functions. For example, mice lacking p21 develop normally [67], presumably because the roles of p21 can be carried out either by p27 or p57. Nevertheless, p21 heterozygous and nullizygous mice are more sensitive to gamma-irradiation [56], consistent with the role of p21 in arresting cell proliferation during periods of genotoxic stress. Mice lacking p27 survive into adulthood, although they exhibit abnormalities that include organ hyperplasia, female sterility, and susceptibility to tumorigenesis [78]. Mice lacking maternal p57 die before birth or very shortly thereafter [79-81]. Thus p57 is the only family member of this group that is required for embryonic development. p57 null mice have large placenta (placentomegaly). Additionally these mice show lens cell hyperproliferation and apoptosis, abdominal muscle defects and cleft palate. These phenotypes correlate well with the restricted expression pattern of p57 (Table 1).

Defects in the placenta associated with the lack of p57 function are explained by the inability of the cells to terminally differentiate into TG cells. In the absence of CDK inhibitor p57, the cells continue to go through mitosis resulting in large placenta. This was also observed when TS cells derived from p57 null blastocysts were differentiated into TG cells [18]. Unlike the wild type TG cells p57 null cells continued to proliferate and did not enter a terminally differentiated state. In contrast with the essential role of p57 in the formation of TG cells, the role of p21 is not essential, apparently because p57 substitute for p21 during differentiation of p21-/- TS cells into TG cells [18]. p57 is also involved in the terminal differentiation of ocular lens cells into secondary lens fiber cells. As with TG cells, loss of p57 also resulted in additional cell divisions in the lens [81].

Interestingly, both the lens cells and cells in the placenta underwent additional cell division in the p27/p57 double knockout animals [82]. This suggests that p27 partially compensates for the role of p57 in the placenta and lens cells. However, whether this compensatory role of p27 applies to TG cells or not is not clear. While p21, p27 and p57 are all expressed in TG cells, only p57 appears to inhibit CDK1 activity in TG cells and is required for endoreduplication. p57 null TG cells express high levels of both p21 and p27, but continue to proliferate. Thus the role of p57 in TG cells can neither be compensated by p21 nor p27. In other tissues, however, the different family members seem to compensate for each other very well. For example both p21 and p57 are expressed at higher levels in skeletal muscles, but neither single mutant animal showed significant defects in muscle differentiation. However, animals that lacked both p21 and p57 showed profound defects in muscle development [83]. Absence of both p21 and p57 resulted in over-proliferation of the muscle cells.

## Conclusion

### p57 Is Often, But Not Always, Associated With Polyploidy

Of the three CIP/KIP CDK inhibitors in mammals, only p27 and p21 have been shown to play a role in regulating mitotic cell cycles. p27 is expressed in all mammalian cells where it is primarily responsible for preventing premature entrance into S-phase, a role that may be common to both mitotic cells cycles and endocycles. p21 facilitates this role and in addition helps to prevent premature entrance into M-phase. The up-regulation of p21 in response to DNA damage arrests cells in G1-phase, revealing that excessive levels of CIP/KIP proteins can block DNA replication. Although more research is needed to clearly define the role of p21 in the differentiation and maintenance of polyploid cells, it appears to be involved in preventing polyploid cells from undergoing apoptosis through suppression of checkpoint pathways.

p57 is expressed at high levels specifically in terminally differentiated cells, most of which are clearly polyploid. In fact, p57 has been shown to be expressed in six of the eight tissues known to produce polyploid cells (Table 1). Surprisingly, however, the ability of p57 to induce endocycles during terminal differentiation of a mammalian tissue appears to be unique to the trophoblast cell lineage. Endoreduplication occurs only when p57 is expressed in TS cells. It is not expressed during endomitosis in MKCs, and its role in myoblast cell fusion is to arrest cells in G0-phase. The role of p57 in the other six tissues where it is expressed is unknown, but its likely role is to arrest cells either in G0 or G2-phase.

### Going Polyploid

The results described above and summarized in Table 1 suggest a unifying view of developmentally programmed polyploidy. First, the essential step is inhibition of cytokinesis, not mitosis. Once cells lose the ability to divide, the presence or absence of p57 and/or p21 determine which of three possible roads to polyploidy is taken. Increasing the level of CIP/KIP proteins above those that occur during mitotic cell cycles arrests cells either in G0 (e.g. myoblasts), or in G2 (e.g. TS cells) or in anaphase (e.g. MKBs). When cells are arrested either in G2 or anaphase, preRC assembly occurs, because CDK activity is suppressed and geminin is degraded. These cells will undergo multiple S-phases without an intervening mitosis and therefore produce a single nucleus. However, if the cellular level of CIP/KIP inhibitors is not significantly elevated in cells that cannot undergo cytokinesis, then acytokinetic mitosis occurs to produce multinuclear cells. The advantage of either acytokinetic mitosis or cell fusion over endoreduplication and endomitosis may simply be that the former do not require uncoupling of checkpoint controls from genome duplication, whereas the later do.

## Competing interests

The authors declare that they have no competing interests.

## Authors' contributions

ZU, CL and MD contributed equally to the research, analysis, organization and writing of this article. MD constructed the figures. All authors read and approved the manuscript.
